# High tibial osteotomy in medial compartment osteoarthritis and varus deformity using the Taylor spatial frame: early results

**DOI:** 10.1007/s11751-011-0123-2

**Published:** 2011-11-10

**Authors:** P. M. Robinson, M. C. Papanna, B. V. Somanchi, S. A. Khan

**Affiliations:** 1 Limb Reconstruction Unit, Department of Orthopaedics, Salford Royal Hospital NHS Foundation Trust, Stott Lane, Salford, Greater Manchester M6 8HD UK; 2 Department of Orthopaedics, Peterborough and Stamford Hospitals NHS Foundation Trust, Bretton Gate, Peterborough, Cambridgeshire PE3 9GZ UK

**Keywords:** Ilizarov, Tibia, Osteotomy, Knee, Osteoarthritis

## Abstract

We report the early results of high tibial osteotomy (HTO) in medial compartment osteoarthritis (OA) and varus deformity using the Taylor spatial frame (TSF). Between October 2005 and April 2007, 9 patients with medial compartment OA and varus deformity underwent TSF application and medial opening wedge HTO. Pre- and post-operative Oxford knee scores, SF-12 and visual analogue pain scores were recorded along with radiographic outcomes. Median follow-up was 19 months (range 15–35). Mean age at operation was 49 years (range 37–59). The median time spent in the frame was 18 weeks (range 12–37). The mean preoperative Oxford knee score was 28.7. This improved to a mean of 35.4 post-operatively (*P* = 0.0142). 6 (67%) patients had a documented pin-site infection. With TKR as an end point, the survival rate of HTOs was 88.9% at a median of 19 months follow-up. This study demonstrates that in selected patients the TSF provides a viable treatment option for performing HTO in medial compartment OA with varus deformity.

## Introduction

The treatment of isolated medial compartment osteoarthritis (OA) in the young or physically active patient is a challenging problem. The rationale for high tibial osteotomy (HTO) in medial compartment OA with varus deformity is to correct varus malalignment and to redistribute load to the non-diseased lateral compartment of the knee.

Tibial osteotomy for OA of the knee was first described by Jackson [1]. Later, closing wedge osteotomy above the tibial tuberosity became widely accepted as a treatment for medial compartment OA [2–4] and its successful use has been reported in several large series [5–13]. Other authors have employed a medial opening wedge osteotomy [14, 15]. The technique has seen renewed interest with the advent of angle-stable locking plates [16, 17].

The use of single bar external fixators and a medial opening wedge has been described by several authors [18–23]. Ilizarov circular fixators have also been utilised to perform opening wedge HTO by distraction osteogenesis [24–27]. The use of the Ilizarov Taylor spatial frame (TSF) (Smith and Nephew, Memphis, Tennessee) offers the advantage of computerised correction planning [28, 29]. We present our experience of this technique.

## Materials and methods

### Patient selection

Patients suitable for HTO had symptomatic medial compartment OA of the knee with varus mal-alignment. The patients were not considered suitable for, or did not wish to undergo, an arthroplasty procedure. This was either because they were felt to be too young or they wished to remain physically active and therefore arthroplasty was not desirable. Patients had a relatively good range of knee movement (>90° flexion and <15° fixed flexion) and no major ligamentous instability. Radiographically patients had medial OA no greater than Ahlbäck grade 2 and had an abnormal mechanical axis deviation (MAD) with varus deformity. Exclusion criteria were lateral compartment OA, symptomatic patellofemoral compartment OA, valgus knees, femoral deformity and obesity with a BMI > 35. Patients were counselled preoperatively about the procedure and gave informed consent.

### Radiographic assessment

Preoperatively patients had weight-bearing anteroposterior (AP) and lateral radiographs centred on the knee, and standard AP and lateral radiographs of the tibia and fibula. Correction planning was performed using long leg standing AP radiographs.

Preoperative radiographic OA grade was recorded using the Ahlbäck [30] classification. Radiographs were used to calculate the pre- and post-operative measurements for the femorotibial angle (mechanical) (FTA_m_), MAD, medial proximal tibial angle (MPTA) and the tibial slope. Measurements of FTA_m_, MAD and tibial slope were made by one author (PMR). MPTA was measured by one author (BVS). The FTA_m_ was calculated using long leg standing AP radiographs. A line was drawn from the centre of the femoral head and from the centre of the tibial surface of the talus to the midpoint of the knee. The medial angle between the intersection of these two lines was recorded as the FTA_m_. An angle of less than 180° was considered varus. The MAD was measured as the distance from the centre of the knee joint to the point at which the lower limb mechanical axis crossed the tibial plateau (mm). The tibial slope was calculated from the lateral radiograph of the knee using the method described by Gunes et al. [26]. The MPTA was measured as the medial angle between the mechanical axis of the tibia and a line parallel to the tibial articular surface.

### Clinical assessment

We collected pre- and post-operative Oxford knee scores (OKS) for each patient. Answers were scored 4–0, with 4 being the score recorded for no symptoms and 0 being recorded for severe symptoms. SF-12 and visual analogue pain score (VAS) were also prospectively recorded. Pin-site infections were graded using the Otterburn classification [31] (Table 1).

**Table 1:** The Otterburn classification of pin-site infections

Grade	Infection responded to
1	Pin-site care
2	Pin-site care and oral antibiotics
3	Reposition of pin, external fixation continued
4	Removal of external fixation with local surgery to control infection
5	Grade 4, with addition of radiological signs of osteomyelitis
6	Chronic osteomyelitis, sequestrum formation

### Correction planning and operative technique

All operations were carried out by a single consultant orthopaedic surgeon specialising in Ilizarov surgery. Preoperative planning was carried out using the online Spatialframe software package (http://www.spatialframe.com).

**Fig. 1:**
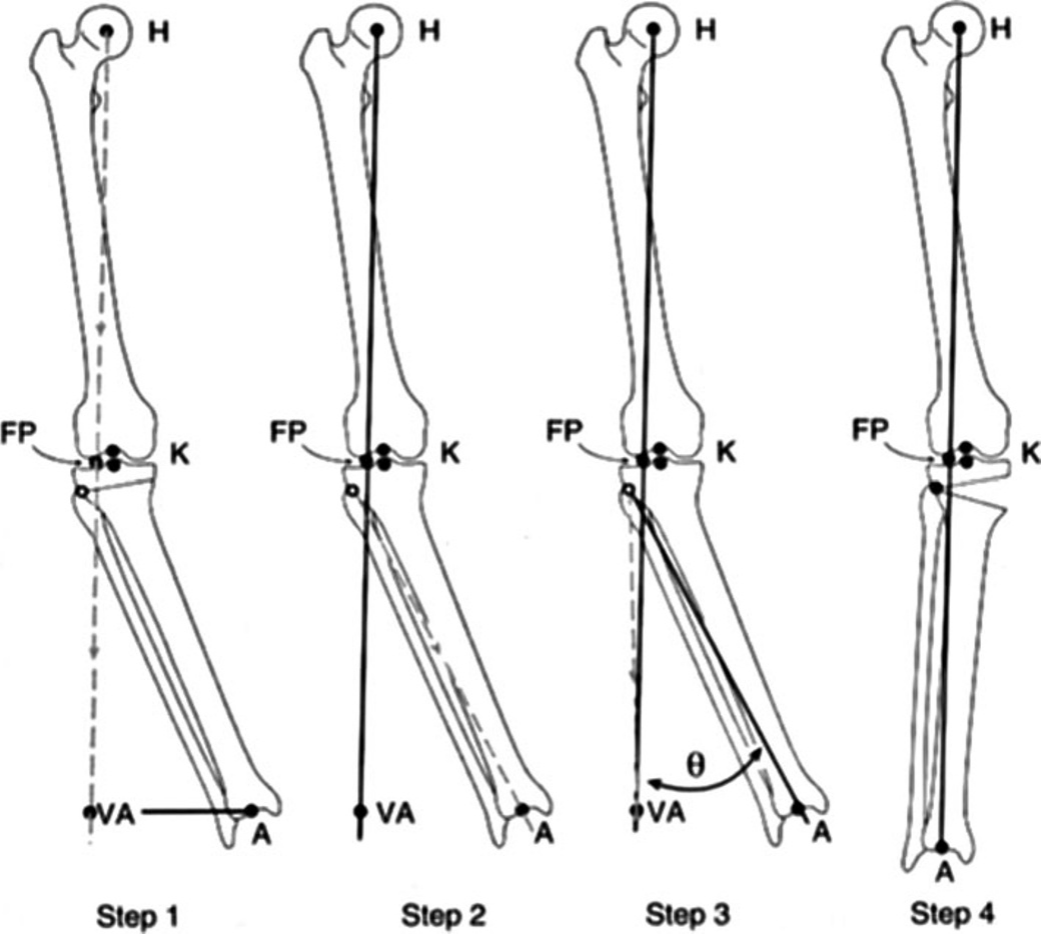
Method of planning the magnitude of the opening wedge [32]. *H* hip, *K* knee, *A* ankle, *VA* virtual ankle, *FP* Fujisawa point. With kind permission from Springer Science + Business Media: Principles of deformity correction. Dror Paley. 2002. Chap. 16, p 493, figure 16-15b

Measurements were taken from long leg standing AP radiographs. In all patients, the varus deformity originated within the knee joint and therefore this was where the centre of rotation of angulation (CORA) was located. The magnitude of the correction to be made was calculated by the technique described by Murphy [32] (Fig. 1). The “new” mechanical axis line was first plotted from the centre of the femoral head passing through the desired point in the medial third of the lateral tibial plateau. This line was continued out to a theoretical point V_A_ (virtual ankle) at the level of the patient’s ankle joint. The line of the intended tibial osteotomy was then drawn. A further line (line 1) was drawn from the centre of the tibial surface of the talus to the anatomical correction axis (ACA) at the lateral edge of the proposed tibial osteotomy. A final line (line 2) was drawn from the ACA to the V_A_ point. The angle (θ) between line 1 and line 2 was the angle of the correction.

**Fig. 2:**
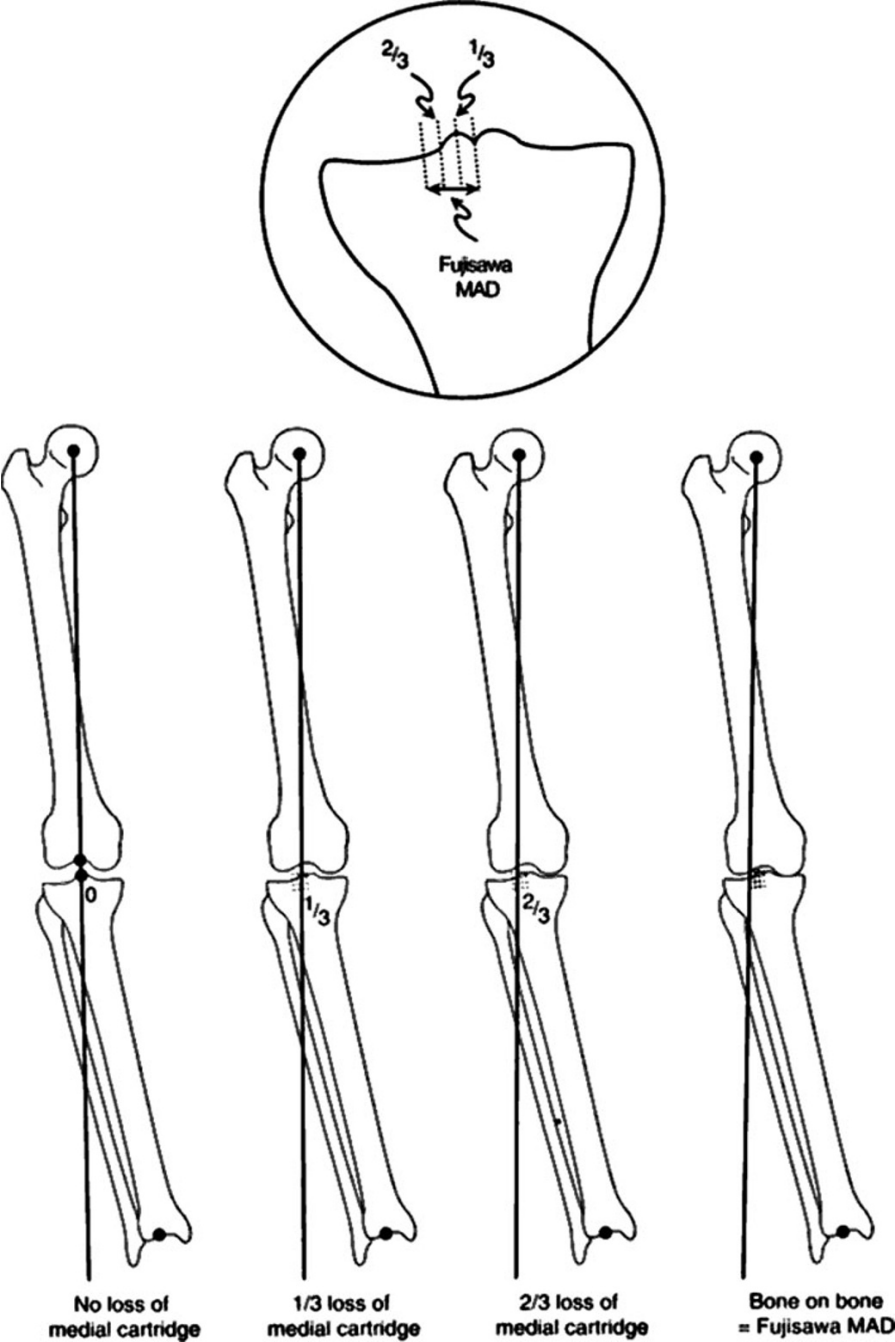
Fujisawa’s point (*top*) and the magnitude of MAD correction made according to the medial compartment joint space [33]. With kind permission from Springer Science + Business Media: Principles of deformity correction. Dror Paley. 2002. Chap. 16, p 481, figure 16-6

The point at which the new mechanical axis was to cross the lateral tibial plateau was decided using the method described by Jakob and Murphy [33] (Fig. 2). The medial compartment joint space was assessed on weight-bearing AP radiographs. If the medial compartment joint space was normal, then the correction goal was a MAD of 0. If the medial compartment joint space was one-third decreased, the correction goal was a MAD of one-third of the Fujisawa point (the Fujisawa point is one-third of the way lateral on the lateral tibial plateau [34]). If the medial compartment joint space was two-thirds decreased, the correction goal was a MAD of two-thirds of the Fujisawa point. If the medial compartment joint space was completely obliterated, the correction goal was a MAD intersecting Fujisawa’s point.

**Fig. 3:**
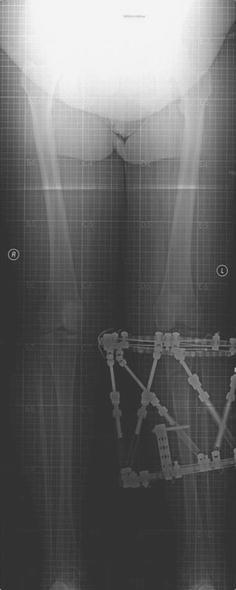
Long leg standing AP radiograph post-correction

The operation was performed under general or spinal anaesthesia. 1.5 g of prophylactic Cefuroxime was given intravenously, and a pneumatic thigh tourniquet was applied but not inflated. The frame was applied using the “rings first” method [28]. The leg was rotated so that the patella was facing forward. Using fluoroscopic imaging, a smooth 1.8-mm wire (Smith and Nephew, Memphis, Tennessee) was advanced across the proximal tibial metaphysis from lateral to medial in the coronal plane, perpendicular to the proximal tibial mechanical axis. The wire was placed at least 15-mm distal to the lateral tibial plateau in order to avoid penetrating the joint capsule. The proximal ring was centred on the leg and the wire tensioned. A 2/3 ring was usually utilised proximally to allow space for knee flexion and leg swelling. The ring was held perpendicular to the mechanical axis of the tibia in the sagittal plane. A second wire was placed anterior to the fibula head exiting the anteromedial part of the tibia. The wire was advanced in a normal fashion, whilst watching the foot for motion. Once the wire tip had crossed through the leg and was tenting the skin, the drill was removed and the wire tapped through. A medial face wire was placed in the same fashion, from posteromedial to exit the anterolateral tibia. The distal ring was then placed with the appropriate strut length taken into account. We predominantly used medium struts. A medial face wire was advanced from posteromedial to anterolateral across the tibia orthogonal to its long axis. Attention was paid to stretching the gastrocsoleus muscles when pushing the wire down to bone. When passing the wires, they were held with a chlorhexidine soaked swab and advanced in a pulsed manner in order to allow heat dissipation. The distal ring was centred on the leg and fastened to the wire. The wire was then tensioned. A coronal plane wire was passed from the midpoint of the subcutaneous surface of the tibia transversely. A third wire was placed between these two wires. A 5-mm half pin was placed proximal to the distal ring in the sagittal plane, medial to the tibial crest. This was inserted through a 1-cm incision in the skin and blunt dissection was performed down to bone. Low-speed drilling was performed using a tissue protector, with frequent pauses and saline irrigation. The distal ring was connected to the half pin using a 5-hole Rancho cube. Occasionally, a further 5-mm half pin was utilised, placed perpendicular to the anteromedial surface of the tibia. This was connected to the distal ring in the same manner. Six struts were attached to the proximal and distal rings, and the strut settings were noted and locked. An oblique metaphyseal tibial osteotomy directed towards the superior tibiofibular joint was made distal to the tibial tuberosity. The struts were disconnected when performing the osteotomy. This was made percutaneously through a 1-cm skin incision over the tibial crest, distal to the tibial tuberosity. A blunt dissection made down through the layers, ending with incision through the tibial periosteum, which was elevated. The osteotomy was made under fluoroscopic guidance using multiple drill holes in the same plane on AP fluoroscopy using a 4.8-mm drill. Pulsed drilling was used and the drill was irrigated with saline to minimise thermal damage. Lateral fluoroscopy was utilised to prevent the drill or osteotome straying into the deep posterior compartment. The osteotomy was completed with a 5-mm osteotome and the struts were re-attached at their previous lengths. The wound was closed with metallic clips and dressed with an opsite dressing (Smith and Nephew Healthcare, Hull, UK). Pin sites were dressed with dressing gauze soaked in alcoholic chlorhexidine (Hydrex, Adams Healthcare, Leeds, UK) held down with a black rubber bung from a 20-ml syringe. Post-operatively patients were allowed to fully weight-bear and they were discharged on the first or second post-operative day in the majority of cases. They were seen in the clinic 1 week later. Post-operative AP and lateral radiographs in the correct rotational alignment were taken centred on the proximal ring. For the purposes of correction planning, the proximal ring was used as the reference ring and the proximal tibiofibular joint was taken as the origin. We measured the distance from the origin to the centre of the reference ring in the coronal, sagittal and axial planes in millimetres from the radiographs. This was entered into the mounting parameters of the TSF online planning software. The required valgus angular correction was entered into the program. The total residual correction mode was utilised. Approximately 3 mm of lengthening was added into the correction to prevent the lateral tibial cortex blocking the angular correction. The structure at risk was the common peroneal nerve and measurements were entered into the program as such. The rate of correction was 1 mm per day. The correction program was initiated between 8 and 10 days post-operatively (latency period). This was given to the patients in the form of a computer-generated colour-coded strut turning schedule. Patients were taught how to perform their own frame and pin-site care and also had access to a nurse-led Ilizarov clinic. They were reviewed on a regular basis in the clinic and the correction was assessed by serial radiographs. When the initial correction program was completed, the limb alignment was assessed by long leg standing AP radiographs (Fig. 3). Once adequate correction had been achieved, the frame struts were locked. When the regenerate bone at the osteotomy site was thought adequately consolidated, the frame was dynamised by sequential release of the struts. When dynamisation was completed, the frame was removed and the patient placed into a below knee walking cast. The pin sites were inspected 1 week later and the cast was exchanged for a lightweight removable cast and worn for a further 5 weeks.

### Statistical analysis

Normally distributed continuous variables are presented as the mean with standard deviation and 95% confidence intervals. Non-normally distributed continuous variables are presented as the median and range. Statistical analysis was performed by one author (PMR) using GraphPad InStat (http://www.graphpad.com/instat/instat.htm). Paired values were compared using the paired, two-tailed Student’s *t* test. Statistical significance throughout is taken at the 5% level.

## Results

Between October 2005 and April 2007, nine patients underwent HTO. The mean age at operation was 48.7 years (SD 7.1). Median follow-up was 19 months (range 15–35). All patients were men. On preoperative radiographic examination, eight knees were Ahlbäck grade 1 and one knee was grade 2. All patients had a varus deformity of the tibia with an abnormal MPTA (Table 2). The median latency was 8 days (range 5–15) and the median duration of correction was 18 days (range 14–36) with 6 patients requiring an additional correction program. The median total angle of correction according to correction program given was 14 degrees (range 10–22). The median length of stay was 2 nights (range 1–4) and the median time in the frame was 18 weeks (range 12–37).

**Table 2:** Mean (SD) MAD, MPTA, FTA_m_ and tibial slope values for the operated and non-operated legs

	Pre-correction		Post-TSF removal		Non-operated leg	
MAD (mm)	28.4 medial	(21.9)	3.9 lateral	(19.6)	9.8 medial	(11.2)
MPTA (°)	77.6	(2.7)	92.1	(2.5)	NA	NA
FTA_m_ (°)	172.3	(5.6)	181.1	(5.2)	177.4	(2.8)
Tibial slope (°)	8.1	(5.1)	7.7	(4.2)	NA	NA

HTOs had an 88.9% survival at a median of 19 months follow-up with total knee replacement as an end point.

**Fig. 4:**
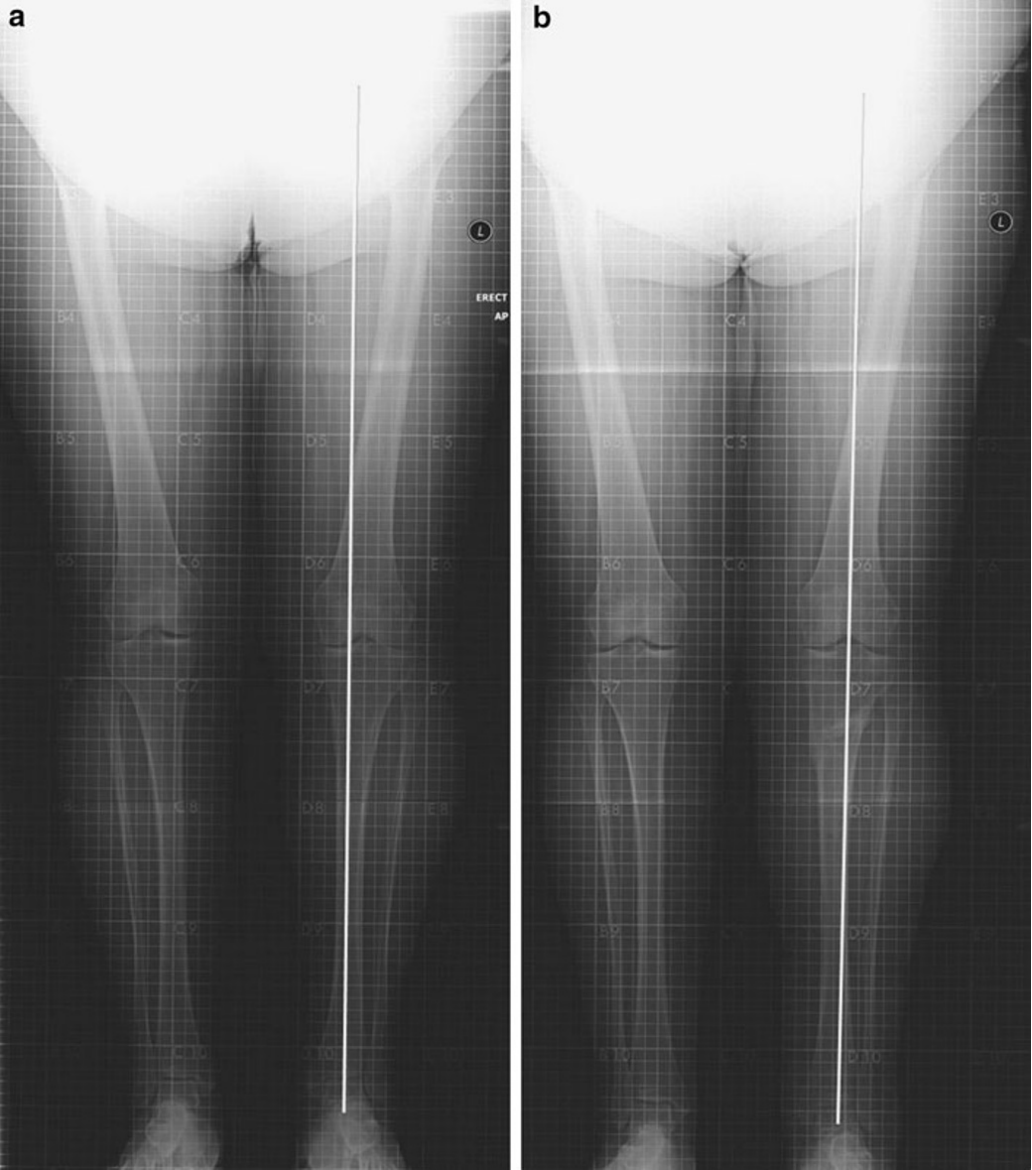
Long leg standing AP radiographs demonstrating mechanical axis. **a** preoperatively, **b** post-correction

Radiographically we achieved a mean FTA_m_ correction of 8.8° (range 4–14) (Fig. 4) and a mean MPTA correction of 14.5° (range 10–21). There was virtually no change in the tibial slope. There was a statistically significant increase in the OKS after HTO (*P* = 0.0142), VAS (*P* = 0.0030) and SF-12 MCS (*P* = 0.0160) (Table 3). At the last follow-up, the OKS had improved in all but one patient (Table 3). The mean OKS of the 8 surviving HTOs was 37.8.

**Table 3:** Mean (SD) and 95% confidence intervals (95% CI) OKS, SF-12 and VAS pre-correction and post-TSF removal at last follow-up

	Pre-correction		Post-TSF removal		*P* value
	Mean (SD)	95% CI	Mean (SD)	95% CI	
OKS	28.7 (10.9)	20.8–36.6	35.4 (12)	26.2–44.7	0.0142
SF-12 PCS component	42.8 (11.0)	33.5–52.0	44.6 (14.0)	32.9–56.3	0.6304
SF-12 MCS component	42.4 (9.7)	34.2–50.5	53.5 (9.4)	45.7–61.4	0.0160
VAS	5.6 (2.1)	3.8–7.4	2.8 (2.4)	0.8–4.8	0.0030

## Complications

6 (67%) patients had a documented pin-site infection (Table 4). The median Otterburn grade was 3 (range 0–4). There were no cases of chronic bone infection.

**Table 4:** Pin-site infections classified by Otterburn grade

Otterburn grade	Number of patients
1	0
2	1
3	4
4	1
5	0
6	0

There was one deep vein thrombosis and one pulmonary embolism that was treated uneventfully. One patient developed a hypertrophic non-union that was diagnosed after TSF removal. The osteotomy united after a further 17 weeks of frame treatment. One patient required a fibular osteotomy due to excessive pain associated with a larger than average correction of 22°.

One patient was not compliant with the strut turning schedule. The osteotomy was fixed with a locking plate and they subsequently underwent total knee replacement at 36 weeks post-frame removal.

## Discussion

We have shown that opening wedge HTO using the TSF is an effective treatment strategy in selected patients with medial compartment OA of the knee and varus deformity. All patients who remained compliant experienced an improvement in their OKS, SF-12 and VAS. It is not clear why patients experienced a larger improvement in the mental component of the SF-12 as compared with the physical component. This may be due to an improvement in psychological morbidity after knee pain had improved.

Recently, there has been renewed interest in HTO as a treatment for medial compartment OA. This is due to several factors, including the increased reliability of angle-stable locking plates [16, 17] and better understanding of the relationship between limb malalignment and OA [35, 36]. Currently, the most widely used method for performing HTO is by open reduction and internal fixation (ORIF). This method can be unforgiving and any under or over-correction can lead to an unsatisfactory result [12, 14, 23]. Methods for increasing the accuracy of correction have been devised [13, 37].

Varus deformity often has a ligamentous component as well as osseous and cartilaginous components. Methods of measuring ligamentous laxity and its effect on deformity have been incorporated into planning algorithms for closing wedge HTO [38]. The TSF allowed soft tissue balance to be taken into account because MAD could be assessed during weight bearing and the correction program was altered accordingly.

HTO using the TSF was first reported by Fragomen et al. [28] citing an average improvement of 16 points in the SF-36 scores. There has been only one detailed study published describing HTO using the TSF for medial compartment OA of the knee [29]. This involved 7 patients who were assessed post-operatively with the lower extremity measure (LEM) questionnaire [39]. The average LEM score in the 5 patients that were followed-up was 94%. The average time spent in the frame was slightly longer than our series at 23 weeks. We report a similar latency period and number of residual corrections. The average FTA_m_ was 8° varus preoperatively and 3° valgus post-operatively compared with 7.7° varus and 1.1° valgus in our study.

All authors agree that some overcorrection is necessary. The exact magnitude is uncertain and some have found that excessive valgus was associated with lateral compartment degeneration [14]. However, others have found no correlation between valgus overcorrection and lateral compartment degeneration [40, 41].

Yasuda et al. [41] recommended that correction to 12°–16° anatomical valgus gave the best results at 10–15-year follow-up. Insall et al. [12] also recommended a larger correction of 10°–14° anatomical valgus. Koshino et al. [9] studied their outcomes at 15–28-year follow-up and recommended at least 10° of anatomical valgus. Vainionpaa et al. [6] noted that the best outcomes were achieved in patients with a post-operative anatomical FTA of 5°–13° of valgus.

Coventry et al. [5] found correcting to ≥8° anatomical valgus gave >90% survival at 5 years and at least 65% survival at 10 years. Contrary to other authors, they did not recommend correction to more than 10° of valgus. There have been fewer published studies involving opening wedge HTO. Hernigou et al. [14] found that the best results were seen when a FTA_m_ of 183°–186° valgus was achieved. Our results show a slight under-correction compared with these studies; however, we did not attempt to correlate outcomes with the amount of correction given. The consequences of under or overcorrection may not be evident in our patients at this early stage of follow-up.

In terms of MAD, Fujisawa et al. [34] found that a post-operative MAD of 30–40% of the lateral tibial plateau width produced the best outcome with regard to the initiation of the natural repair of articular cartilage and meniscal lesions. Dugdale and Noyes [38] recommended an ideal post-operative weight-bearing line (WBL) of 62–66%, corresponding to a FTA_m_ of 3°–5° of valgus. Again, our results show a slight under-correction compared with these figures.

Previous studies of opening and closing wedge HTO with internal fixation have found significant changes in the tibial slope [42–44] altering the normal knee biomechanics. We found very little difference in the tibial slope. This confirms the results of earlier studies of HTO using the TSF [29].

One further advantage of the TSF is that it enables an osteotomy below the tibial tuberosity, minimising any alterations of patellofemoral mechanics [20, 45]. However, we cannot confirm whether this was clinically beneficial in our patients.

The use of a circular fixator to perform HTO was first published by Catagni et al. [24]. They used the Ilizarov fixator and an osteotomy distal to the tibial tuberosity. The mean duration of treatment was 85 days. They reported significant pain relief in 54 of 55 osteotomies at a follow-up of 12–60 months.

There has been one previously published study comparing opening wedge HTO by Ilizarov circular fixator with a Coventry closing wedge [27]. The authors reported that patients undergoing Ilizarov correction had a significantly greater decrease in pain and increase in function at a mean follow-up of 28 months. The magnitude of correction performed in the Ilizarov group was similar to that of our study. The Ilizarov patients spent an average of 12.9 weeks in the frame and there was no difference in osteotomy healing rates. The time spent in the frame in these two studies was much shorter than our series. Six of our patients required an additional TSF program to achieve adequate correction, but it is unclear whether this was due to our method of correction planning.

The disadvantage of using an external fixator is the frame-related morbidity, which can lead to non-compliance. Indeed, our one reported failure was due to this. In terms of pin-site infections, our study was comparable with the results of others. Magyar et al. [23] reported 18 pin-site infections classified as Otterburn grade I or II in 15 patients. Viskontas et al. [29] and Adili et al. [27] reported a similar rate and severity of pin-site infections to our study (71% and 70.5% versus 67%).

Our study has several limitations. There was no randomisation, control group or alternative treatment group. Due to the relatively unique nature of this treatment and the narrow inclusion criteria, our study group was small. This does not allow for any more complex statistical analysis.

In conclusion, this study demonstrates that in selected patients the TSF provides a viable treatment option for performing HTO in medial compartment OA with varus deformity. Correct patient selection and more importantly thorough pre-operative counselling are essential in order for a successful outcome due to the rate of frame-associated morbidity experienced with this treatment.
